# Parental Perspectives on Pediatric Surgical Recovery: Narrative Analysis of Free-Text Comments From a Postoperative Survey

**DOI:** 10.2196/65198

**Published:** 2024-12-20

**Authors:** Jessica Luo, Nicholas C West, Samantha Pang, Julie M Robillard, Patricia Page, Neil K Chadha, Heng Gan, Lynnie R Correll, Randa Ridgway, Natasha Broemling, Matthias Görges, Pediatric Pain Prediction Collaboration

**Affiliations:** 1 Research Institute BC Children’s Hospital Vancouver, BC Canada; 2 Department of Anesthesia BC Children’s Hospital Vancouver, BC Canada; 3 Division of Neurology Department of Medicine The University of British Columbia Vancouver, BC Canada; 4 BC Children’s and Women’s Hospital Vancouver, BC Canada; 5 Division of Pediatric Otolaryngology-Head and Neck Surgery Department of Surgery The University of British Columbia Vancouver, BC Canada; 6 Department of Anesthesiology, Pharmacology & Therapeutics The University of British Columbia Vancouver, BC Canada

**Keywords:** narrative analysis, qualitative data, family feedback, narrative feedback, pediatric surgery, perioperative care, pain management, surgical recovery

## Abstract

**Background:**

Qualitative experience data can inform health care providers how to best support families during pediatric postoperative recovery. Patient experience data can also provide actionable information to guide health care quality improvement; positive feedback can confirm the efficacy of current practices and systems, while negative comments can identify areas for improvement.

**Objective:**

This study aimed to understand families’ perspectives regarding their children’s surgical recovery using qualitative patient experience data (free-text comments) from a prospective cohort study conducted within a larger study developing a postoperative-outcome risk stratification model.

**Methods:**

Participants were parents or guardians of children aged 0-18 years who underwent surgery at a pediatric tertiary care facility; children undergoing either outpatient or inpatient procedures were eligible to be enrolled. Participants with English as a second language were offered translational services during the consent process and were included if any family member could translate the surveys into their preferred language. Participants were ineligible if they and their families could not understand English or the child had a neurodevelopmental disability. Perioperative data were collected from families using web-based surveys, including 1 preoperative survey and follow-up surveys sent on postoperative days 1, 2, 3, 7, 15, 30, and 90. Surveys were completed until the family indicated the child was fully recovered or until postoperative day 90 was reached. Follow-up surveys included opportunities to leave free-text comments on the child’s surgical experience.

**Results:**

In total, 91% (453/500) of enrolled families completed at least 1 postoperative survey; 53% (242/453) provided at least 1 free-text comment and were included in the presented analysis, based on a total of 485 comments. The patient’s age distribution was bimodal (modes at 2-3 and 14-15 years), with 66% (160/242) being male. Patients underwent orthopedic (60/242, 25%), urological (39/242, 16%), general (36/242,15%), otolaryngological (31/242, 13%), ophthalmological (32/242, 13%), dental (27/242, 11%), and plastic (17/242, 7%) surgeries. Largely positive comments (398/485, 82%) were made on the recovery and clinical care experience. A key theme for improvement included “communication,” with subthemes highlighting parental concerns regarding the “preoperative discussions,” “clarity of discharge instructions,” and “continuity of care.” Other themes included “length of stay” and “recovery experience.” Feedback also suggested survey design amendments for future iterations of this instrument.

**Conclusions:**

Collecting parental recovery feedback is feasible and valued by families. Findings underscored the significance of enhancing communication strategies between health care providers and parents to align expectations and support proactive family-centered care. Our postoperative surveys allowed families to provide actionable suggestions for improving their experience, which may not have been considered during their hospital encounter. Our longitudinal survey protocol may be expanded to support continuous quality improvement initiatives involving near-real-time patient feedback to improve the health care experience of patients and families.

## Introduction

### Background

Narrative experience data can be valuable in informing health care providers and play a crucial role in shaping health care policies [1,2]. In pediatrics, the parents, guardians, or caregivers are core health care team members. Qualitative research may provide rich insights into patients’ and their families’ preoperative and postoperative experiences [3-6] Developments in text analytics, including natural language processing, have allowed for increasingly efficient transformation of unstructured, qualitative data into interpretable insights for health care quality improvement (QI) at systematic, institutional, and departmental levels [7,8]. Analyzing patient-generated experience data has many benefits, such as informing clinical decision-making, tracking medication adherence, and understanding experience sentiment [8].

### Narrative Research at Our Institution

A 2011-2018 narrative analysis of patient experience data from our pediatric tertiary care facility identified high satisfaction levels with their care and positive experiences with providers. Areas for improvement were found in various domains, including pain management and postoperative complications [9]. Same-day or outpatient surgery is considered beneficial for both health care systems and families, but discharge on the same day may not always be feasible, often due to surgical complexity and postoperative pain management requirements; however, unanticipated hospitalization may be avoided by optimizing surgical, anesthetic, and analgesic practices [10]. In 2020, BC Children’s Hospital (BCCH) implemented 2 strategies to improve outcomes and communication at discharge, that are (1) families receive take-home comfort brochures with procedure-specific guidance on medication and alternative pain management strategies after a same-day or outpatient surgery [11] and (2) nurses make postoperative follow-up (POFU) calls to families of children undergoing outpatient procedures within 24 hours of discharge to provide more information or resources, if necessary [12]. Until this study, our institution had not conducted a narrative analysis of patient experiences after implementing these 2 strategies.

### Study Rationale

Despite modern health care practices, postoperative pain management and surgical recovery can be complicated for pediatric patients. Of all, 1 prevalent adverse consequence in the pediatric population is chronic postsurgical pain (CPSP) [13,14]. Patients with CPSP may experience a range of negative consequences, including a decreased quality of life, decreased trust in the health care system, and increased opioid usage [15-17]. Risk factors for pediatric CPSP are suspected to be influenced by a range of biopsychosocial factors [14,16].

Recognizing the significant impact and need for further research on CPSP within the pediatric population, our research team is developing a pediatric pain risk prediction (PPRP) system [18] to identify children at higher risk of significant postsurgical pain based on factors known at the time of surgical booking [19], along with a risk communication tool [20] to help families make more informed decisions about, and better preparation for, their child’s surgery. To support the development of PPRP models [18], we conducted a prospective cohort study, which collected (1) preoperative data to identify potential risk factors, as well as (2) postoperative experience and outcome measures to characterize the quality of recovery from surgery. Collecting these data allowed us to revisit narrative experience data at our institution, as the postoperative surveys captured free-text comments by the caregivers of pediatric patients.

### Study Aims

The objectives of this qualitative analysis of free-text comments were to (1) evaluate current pediatric perioperative practices and recovery experiences from the parental perspective and (2) identify opportunities to optimize our study survey scheme for future research or QI implementations.

## Methods

### Study Design

The PPRP study collected quantitative data from families, which included a pediatric patient and their parent or guardian, using (1) a preoperative questionnaire and (2) a series of postoperative questionnaires. Wood et al [19,20] describe study design and modes of delivery in detail. In short, the data collection approach, including survey design, was codeveloped with parents of children who had previously undergone surgery, adults with lived pediatric surgical experience, and clinicians who work at BCCH. The REDCap (Research Electronic Data Capture; Vanderbilt University) tool [21] facilitated data collection. The study had 3 arms based on patient age groups: 0-4, 5-12, and 13-18 years; these were based on our use of validated health measures from the PROMIS (Patient-Reported Outcome Measurement Information System) [19,22]. We aimed to maximize self-report, where possible. All questionnaires for patients aged 0-12 years were completed by a parent or guardian. Preoperative questionnaires for children aged 13-18 years were completed by both the adolescent patient and a parent or guardian; however, postoperative “outcomes” questionnaires were completed by the adolescent patient only, and postoperative “experience” questionnaires, which allowed for free-text comments, were completed by a parent or guardian only.

Postoperative questionnaires were sent on postoperative days (PODs) 1, 2, 3, 7, 15, 30, and 90. Surveys were completed until they deemed their child or themselves fully recovered or upon reaching the final survey on POD 90. Each postoperative questionnaire collected patient-reported outcome measures and patient-reported experience measures [19]. Our findings are reported following the Standards for Reporting Qualitative Research [6].

#### Free-Text Field

**Figure 1:**
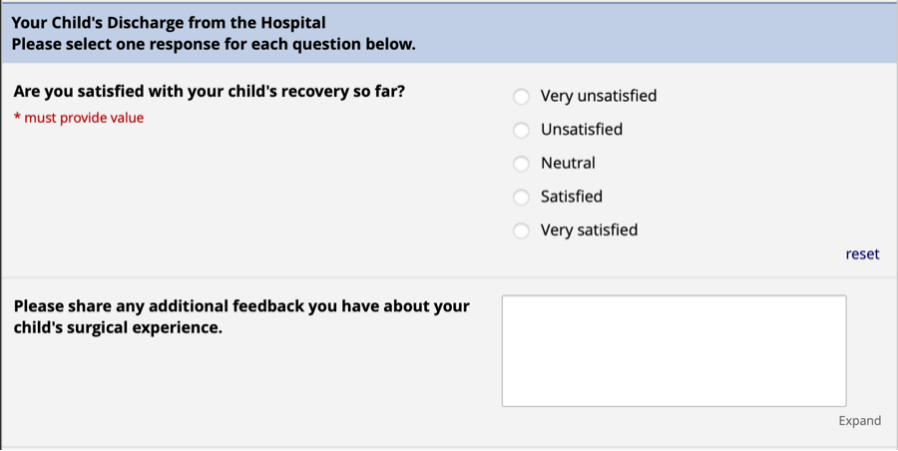
A screen capture of the pediatric pain risk prediction survey question asking about caregivers’ satisfaction with their child’s recovery and a section for free-text comments.

At the end of each parental postoperative questionnaire, parent or guardian participants were asked to provide free-text comments based on the following prompt: “Please share any additional feedback you have about your child’s surgical experience” (Figure 1). Providing free-text comments was optional. There were no character or word limits. Handwritten comments from paper surveys were able to continue into the margins. Handwritten comments from paper surveys were transcribed into REDCap. No comments were collected for the 13-18-year age group during PODs 1-3, as parent or guardian participants were only sent postoperative questionnaires for this age group on PODs 7-90.

### Setting and Participants

BCCH is a pediatric tertiary care facility with approximately 9000 surgical procedures annually. The PPRP study targeted 500 families, 375 with children aged 12 years or younger and 125 aged 13 years or older, who were booked for surgery at BCCH.

#### Inclusion Criteria

Families were included if the patient was 18 years or younger and booked for an outpatient or inpatient surgical procedure within the following specialties: dentistry, otolaryngology, urology, ophthalmology, orthopedics, plastic surgery, and pediatric general surgery. Patients having multiple procedures under a single anesthetic were included. Families were included if survey translation was not required or if a family member could translate the survey for the participants.

#### Exclusion Criteria

As the main study was primarily gathering data for postoperative pain trajectories and recovery outcomes, families were excluded if the patient was undergoing a multi-stage procedure (over different days) or had a neurodevelopmental disability (such as a global developmental delay) to reduce analytic complexity.

#### Recruitment

Research assistants approached families at BCCH during their preadmission clinic visit or in the anesthetic care unit before their surgery. Non-English speakers were recruited using spoken language interpreting services offered by the Provincial Health Services Authority. During enrollment, research assistants informed families of the option to provide free-text feedback about their surgical experience within each postoperative survey.

### Ethical Considerations

Ethical approval was obtained from the Children’s & Women’s Health Centre of British Columbia Research Ethics Board, University of British Columbia (H21-02788; date of approval August 05, 2022; principal investigator MG). Each parent or guardian provided written informed consent to participate, and each child aged 7 years or older provided written assent. Each participating family was provided with a unique participant identifier, and no identifying details were retained in the analyzed deidentified dataset. Families were remunerated with an electronic gift card valued at approximately US $15 after completing all postoperative questionnaires, up to the final survey or when they reported their child was fully recovered.

### Data Acquisition

Data capture requirements and delivery modalities have been described previously [19]. Data collection began in August 2022 and finished in January 2024, when the 500th family completed all surveys. Families received surveys electronically (links sent through SMS text message or email) or on paper. Participants were asked to complete 1 preoperative and up to 7 postoperative surveys. A researcher team member added perioperative data, including surgical service, from our hospital’s electronic medical records system (Cerner Corp).

### Data Analysis

Quantitative data were summarized using R software (version 4.3.1; R Core Team). Qualitative data were analyzed in NVivo (v14.23.0, Lumivero) using thematic analysis by 2 researchers with no relationship to any patients or families nor direct involvement in their medical care (JL and NCW). The first researcher (JL) inductively coded the raw responses by initially reading the free-text data repeatedly (familiarization) [23]. Preliminary codes were sorted and synthesized by JL, combining similar concepts into key themes and subthemes (thematic charting). Through thematic content analysis, narrative data was reduced to quantitative measures related to the key thematic categories. Coded free text within comments was categorized as positive or negative. The second researcher (NCW) independently reviewed the comments, verified the inductive codes, and then deductively coded the comments using predetermined categories “hospital,” “patient,” “survey,” “actionability,” and “sentiment.” Both researchers discussed their coding before and after NCW had independently reviewed the comments. Together, JL and NCW then compared interpretations and adjusted the coding frameworks accordingly to ensure that the concepts were consistent and that all key themes were accounted for. Coded quotes were finalized into agreed-upon themes, subthemes, and sentiments.

Blockquotes are identified by italics and indenting. The language used in comments was preserved as much as possible to maintain the intended context. Square brackets indicate changes for spelling, grammar, and deidentification.

Key emerging themes were used to inform relevant health care departments (anesthesia, nursing, and surgery) and help identify future QI initiatives. Based on departmental feedback on the free-text feedback, we generated site-specific recommendations for future iterations of this survey scheme.

## Results

### Participation

The number of potential candidates at BCCH was approximately 5900 in 1 year. The overarching study recruited 500 families; 375 patients aged 0-12 years and 125 patients aged 13-18 years. Patients underwent surgery between August 2022 and August 2023. Data were collected up to January 2024. In total, 91% (453/500) of enrolled parents or guardians completed at least 1 postoperative survey and were deemed “active participants.” Of these, 53% (242/453) provided at least 1 free-text comment and were deemed “included participants” for this qualitative analysis. Of the 242 included participants, 122 (50.4%) wrote 1 postoperative comment, and 120 (49.6%) wrote more than 1 comment. The total number of comments received was 485 (Table 1).

**Table 1:** Distribution of recruited (enrolled in the study), active (completed at least 1 postoperative survey), and included (made at least 1 free-text response) participants by patient age and surgical service.

Participant characteristics			Recruited, n		Active, n (% of recruited)		Included, n (% of active)
Total			500		453 (90.6)		242 (53.4)
**Age group (years)**							
	0 to 4	191		172 (90.1)		89 (51.7)	
	5 to 12	184		163 (88.6)		85 (52.1)	
	13 to 18	125		118 (94.4)		68 (57.6)	
**Surgical service**							
	Dentistry	50		47 (94)		27 (57)	
	Otolaryngology	68		58 (85)		32 (55)	
	General	70		64 (91)		36 (56)	
	Ophthalmology	86		79 (92)		31 (39)	
	Orthopedics	102		95 (93)		60 (63)	
	Plastic surgery	40		33 (83)		17 (52)	
	Urology	84		77 (92)		39 (51)	

### Participant Characteristics

**Figure 2:**
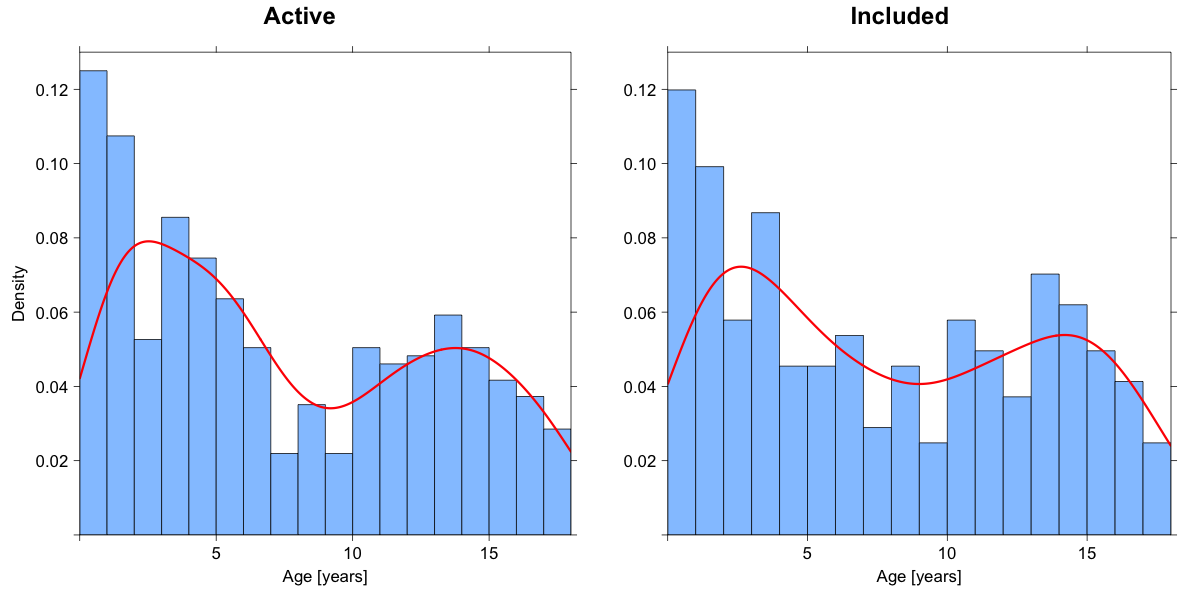
Histogram and density plots of the age distribution of active versus included families. The red line represents the density curve. A bimodal pattern is shown in both active and included participants, with 2 peaks: around the ages of 2-3 years and 14-15 years.

Of the 242 included participants, 185 (76%) were mothers, 54 (22%) were fathers, and 3 (1%) were guardians. Parental self-reported race is presented in Table 2. The median household income range, originally recorded in Canadian dollars, was approximately US $75,000-US $112,500; IQR approximately US $37,500-US $75,000 to US $112,500-US $150,000. A total of 26% (62/235) of caregivers had been diagnosed with or sought treatment for anxiety, and 11% (27/235) had been diagnosed with or sought treatment for chronic pain. Response to parental anxiety and parental chronic pain was omitted by 3% (7/242) of participants. The median age of patients was 7 (IQR 3-13) years. However, the age distribution of the participants was bimodal; the first mode centered around 2-3 years old, while the second mode centered around 14-15 years old. The age distribution of included participants did not differ from that of active participants (Figure 2). Two-thirds of patients (160/242) were male. Outpatients comprised 75% (182/242) of the included participants.

**Table 2:** Distribution of recruited and included participants by parental self-reported race, a demographic collected in the preoperative surveys only^a^. Metro Vancouver census data are provided for reference.

Self-reported race	Total recruited (N=500), n (%)	Included for qualitative analysis (n=242), n (%)	2021 census for Metro Vancouver [24] (n=2,607,015), %
Black	5 (1)	1 (0.4)	1.26
East Asian	46 (9.2)	16 (6.6)	22.03
Indigenous	21 (4.2)	10 (4.1)	2.39
Latin American	9 (1.8)	5 (2.1)	1.97
Middle Eastern	8 (1.6)	3 (1.2)	3.32
South Asian	40 (8)	23 (9.5)	13.81
Southeast Asian	21 (4.2)	12 (5)	7.15
White	239 (47.8)	139 (57.4)	42.01
Another race category	14 (2.8)	8 (3.3)	0.63
Multiple race categories	31 (6.2)	17 (7)	5.44
No answer	66 (13.2)	8 (3.3)	—^b^

^a^Survey responses were grouped into categories using the Guidance on the Use of Standards for Race-Based and Indigenous Identity Data Collection and Health Reporting in Canada [25].

^b^Not applicable.

The median pain score when commenting was 2 (IQR 1-5) out of 10. Based on the number of completed postoperative surveys, the median recovery time was 22.5 (IQR 7-90) days, with the final pain score being 2 (IQR 0-4) out of 10. Only 1 respondent within the included participants indicated their child was not fully recovered by POD 90; however, 31% (74/242) did not respond, and their postoperative surveys were not completed up to full recovery or POD 90.

### Qualitative Findings

#### Overview

Participants provided largely positive comments (398/485, 82%) on their child’s recovery and clinical care experience, expressing gratitude and appreciation for the physicians, nurses, and other hospital staff members who contributed to their surgical experience. Helpful and reassuring family-provider interactions were key reasons for a positive surgical experience and satisfaction with BCCH. A total of 3 themes for improvement arose from the free-text comments, that are “communication,” “length of stay,” and “recovery experience.”

#### Theme 1: Communication

Effective communication was vital across the entire surgical experience for families. Many participants commented positively on their care and the communication of our staff. Some notable improvements could be identified at 3 periods—preoperatively, upon discharge, and at home.

#### Subtheme 1a: Preoperative Communication

The conversations before surgery were found to be defining moments for families. Around 8% (20/242) of participants commented on their preoperative discussions; most (14/20) were positive in sentiment, yet some (6/20) described the experience as being negative or substandard.

The pre-op discussions with all members of the healthcare team (ophthalmologist, anaesthesiologist, nursing staff, child life, booking staff, etc.) were all very helpful. The staff are amazing, kind and have made the surgical experience for both my son and I stress-free! Thank you!

Respondent for a child aged 4 years undergoing strabismus repair

Was so impressed with the friendly staff, positive attitudes. Everyone took their time explaining the procedure not just to us but my child as well, one key point [to note] was that everyone came to see him so when he went to the operative room there were no new faces and my child was not uncomfortable. Who ever needed to do things with him talked their way through it.

Respondent for a child aged 7 years undergoing multiple teeth extraction and intraoral restoration

[We wished] to be educated [on the] postop expectations & rehabilitative process [in] preop.

Respondent for a child aged 13 years undergoing anterior thoracoscopic thoracic tether correction

Another participant described the equipment and supplies they used during their recovery and made this suggestion for preoperative preparedness:

…other patients might benefit from a list of possible items to make recovery easier for families - our favourites are a more robust cast slipper/shoe, shower covers, and a call button which you can plug in anywhere - these are all available on [an online store] for a reasonable price and would make coming home to recover easier if they were purchased prior by the family.

Respondent for a child aged 14 years undergoing Achilles tendon lengthening

#### Subtheme 1b: Discharge Instructions

Participants’ comments on their recovery instructions and pain management plans (26/242, 14%) were mixed in sentiment (12/26, 46% positive and 14/26, 54% negative), emphasizing the importance of accurate and clear communication at discharge for patient outcomes.

Everything was explained perfectly, and I felt comfortable taking care of him at home. The staff were amazing and took the time to go over everything and what I should expect in the following days.

Respondent for a child aged 4 years undergoing inguinal hernia repair

We did not receive any paperwork related to the post-operative instructions specifically for the NUSS procedure that our son had. Other than conversations with the surgeon, all of the information I have about what he should and should not do after the surgery was found via research online from other hospitals that perform the NUSS procedure. For pain medication, we received specific instructions about dosage. That was very clear, but I was unclear on when we should stop giving medication or how best to wean our son off of the pain medications. We had a verbal conversation with the surgeon about follow up appointments, but no written instructions were provided.

Respondent for a child aged 16 years undergoing minimally invasive repair of pectus excavatum

Discrepancies between physicians, nurses, and written instructions were noted as a cause of confusion by families. These participants criticized:

The only frustrating part is that (it seems to often be the case) the doctor will give you different guidance than what the discharge nurse gives you, and both of these are different from the pamphlet you get. I default to trusting the doctor, but it’s hard to know which is right.

Respondent for a child aged 6 years undergoing inguinal hernia repair

Surgery went very well. Day after surgery, however, was very stressful as 3 different teams had 3 different plans and were not on the same page. Ex. Surgeon said going home my child would be at hospital for 3-4 days but APS woke her up the next day to get her ready to go home. Then physio team came and said she needed to be up and walking around the ward 3 times per day, then surgery fellow came and said she should not be walking at all and leg had to be still. The mixed messages caused stress.

Respondent for a child aged 17 years undergoing medial patellofemoral ligament and reconstruction, tibial tubercule osteotomy with nerve block

Some participants made actionable suggestions on how their discharge process could have been improved:

I would appreciate post-care instructions being given out electronically as opposed [to] paper as with children around papers get damaged or go missing.

Respondent for a child aged 11 years undergoing strabismus repair

…he woke up quickly, and the aftercare instructions given to us were given while he was awake, which made it very hard to concentrate on what the nurses were saying. We would have preferred getting instructions before he was awake so we could focus on instructions and not have to split our attention.

Respondent for a child aged 8 years undergoing umbilical hernia repair

#### Subtheme 1c: Continuity of Care

In total, 14% (33/242) of comments were regarding continuity of care. Most comments were positive in sentiment (20/31, 61%); health care follow-ups and continued care through nursing support were valued as helpful components for pediatric postsurgical recovery at home. Providing families with a number they could call if they had further questions or pressing concerns was especially appreciated. However, around 39% (13/33) were negative in sentiment; some asked for an alternative contact when the person they were given was unavailable, while others were not given any phone number.

Follow-up appointments have been very helpful to determine recovery progress. Being able to reach out to the clinical nurse (by phone/email) is a huge advantage, especially when we are not sure what to do about pain management.

Respondent for a child aged 18 years undergoing intramedullary nail insertion

One participant commented on their challenges with continued care at POD 90:

The surgery was great. Past the operative follow up with the surgeon, there is no continued care from the surgeon or hospital. I have found a physiotherapist and have gotten help that way, but neither the physio nor leg brace people at [orthotic service company] (great place and people) are equipped with answers for recovery - how to get stronger after muscle wasting, what kind of physio, how soon to start walking without braces, how to mobilize ankles, etc. There is a big gap in care post surgeon check-up and afterwards.

Respondent for a child aged 14 years undergoing Achilles tendon lengthening

Nursing support was highly valued by families:

Very pleased with how my son and I were able to contact the nurses if we had any questions or concerns. If they didn’t know the answer, they advised us who we should call or phoned us back once they found out the information!

Respondent for a child aged 17 years undergoing circumcision

Some participants were given options for continued supplementary care at home but were challenged when they needed support outside typical work hours.

It was difficult to reach the follow-up team for after-hours questions. The instructions I was given were not correct, and the front desk refused to put me through to anyone.

Respondent for a child aged 15 years undergoing toe nail excision

I had one phone number for support, but the nurse was away on vacation. Would have been nice to have another nurse’s phone [number]. Also, hours of service are 9 am to 4 pm. Would have been nice to have evening hours.

Respondent for a child aged 11 years undergoing circumcision

#### Theme 2: Length of Stay

A notable theme that arose was families’ inclinations toward having a longer length of stay (7/242, 3%). The first hours upon emergence from anesthesia and the first few days at home were reported with the greatest negative sentiment (6/7, 86%). A haste for discharge made some caregivers uncomfortable, making them believe they should have stayed longer.

We feel our child would have benefited from longer observation after surgery, as we were required to return later that afternoon. We felt rushed out of the hospital.

Respondent for a child aged 1 year undergoing circumcision and meatoplasty

I felt my child was not ready to leave her bed with the way she was out of anesthesia. I know there is a nurse shortage, and they were efficient and good at their jobs; I felt almost made to leave while my daughter was still under the effects of anesthesia, and I was uncomfortable leaving with her.

Respondent for a child aged 11 years undergoing extraction of teeth

The only positive comment regarding this subtheme was from a parent who wrote of their appreciation for a longer length of stay:

I am so happy that my son stayed two nights in the hospital as I don't know if I could have handled it on my own at home.

Respondent for a child aged 15 years undergoing an ulnar osteotomy

#### Theme 3: Recovery Experience

Families largely gave updates on their child’s perioperative experiences and daily progress during the postoperative period (38/45, 16%). Overall, participants reported diminished pain in their child throughout the survey, and almost all returned to normal within 3 months. Of those who commented on their recovery journey, around half were positive in sentiment (20/242, 53%) and half were negative (18/242, 47%). Caregivers who were given clear, realistic expectations of their child’s pain and recovery experience seemed to appreciate the preparation.

My son has healed quicker than I anticipated. Still recovering and healing, but every day is much better than the one before.

Respondent for a child aged 3 years undergoing inguinal hernia repair

Still not feeling well post-surgery so is trying to rest instead of being active.

Respondent for a child aged 12 years undergoing adenoidectomy and bilateral myringotomy with tympanostomy tubes

Furthermore, 2 participants who underwent the same surgery had similar comments regarding their pain expectations:

She has much more pain than we anticipated, and we have a hard time understanding what is good pain and what to worry about.

Respondent for a child aged 15 years undergoing arthroscopic anterior cruciate ligament reconstruction with a nerve block

I expected being sent home the same day meant her pain would be less significant. I did not plan for a sufficient leave from my work or enough additional support for my family.

Respondent for a child aged 14 years undergoing arthroscopic anterior cruciate ligament reconstruction

#### Additional Findings: Survey Design

Families used the free-text section to contextualize their preoperative or postoperative survey responses. Participants explained their assumptions regarding any questions within the postoperative surveys, and many clarified their response to “Has your child completely recovered from surgery and is free of pain and has returned to their normal activities?” located immediately after the free-text comment field. Feedback contained suggestions on survey answer options and language clarification for select questions, but no comments were explicitly given on the survey modality (electronic or paper), length of individual surveys, and survey fatigue.

My child had his operation yesterday, so when you ask if he has felt pain in the last 7 days, I assume you meant before his operation. Also, my child is 1.5 years old, so when you ask “Can he stand on his tip-toes,” I had to answer “Not at all” simply because he was unable to do that before his operation.

Respondent for a child aged 1 year undergoing hypospadias repair

There needs to be a spot for “not applicable” for some of these questions. Many didn’t apply to my son.

Respondent for a child aged 8 years undergoing umbilical hernia repair

I want to comment here about the preoperative survey about the time it takes to get to BC Children’s and the follow up appt. We are from [Prince George] so it’s 10 hours driving to get here or a 1.5-hour flight. From the hotel we are staying at, it’s about a half hour to BC Children’s and about 15 minutes to the follow-up appointment. [I found the questions] challenging to answer because they weren’t reflective of out-of-town patients.

Respondent for a child aged 12 years undergoing posterior vitrectomy and retinal photocoagulation with laser

### Site-Specific Recommendations for Future Implementation

Following a presentation of our thematic analysis and discussions with anesthesiology, nursing, and surgical groups, we developed a set of recommendations for our institution that addresses key points in our preoperative information, perioperative communication, discharge, and follow-up processes (Table 3). While the majority of families commented very positively about their experience, these QI recommendations may be implemented to optimize care for all patients.

**Table 3:** Site-specific recommendations based on family and departmental feedback.

Item	Explanation
Provide physician-recommended, specific, digital resources for families	Throughout the surgical experience (booking to full recovery), resources and instructions for families should be physician-recommended; specific to the patient, procedure, and hospital; and available electronically. Health care providers should collaborate routinely to ensure resources are comprehensive, up-to-date, and procedure-specific. Collaborative sessions may be facilitated by the BCCH^a^ PainCare360 team [26].
Ensure timely communication	To reinforce proactive preparedness, health care providers should optimize the timing of information and reminders to families about their surgery, recovery, and pain management strategies. For families who had their preoperative discussion many weeks or months before their surgery, a 1-week reminder may allow them to gather necessary care supplies, arrange postoperative therapies, and coordinate any unique family needs. Where possible, families should have the option to receive postoperative recovery information before the surgery instead of during discharge.
Promote empowerment and understanding at discharge	During discharge, nurses should provide opportunities for families to consolidate their understanding of their child’s postoperative care plan, for example, by allowing families to explain their discharge instructions back to the health care providers. If any instructions between nurses and doctors are contradictory, families should have time to clarify while they are at the hospital. If a family is hesitant about their discharge from the hospital, nurses should explain the rationale for the discharge, reassure the family, address any concerns, and reiterate the range of postdischarge supports available.
Continue to listen	The BCCH postoperative follow-up or POFU^b^ program [12] has been helpful for families and should be continued. We should also revise and reimplement the perioperative self-report survey piloted in this study and send it to all families to supplement the POFU program so that health care professionals can be updated on their patients’ outcomes and address concerns in near-real-time. Survey questions should be revised based on participant feedback and expanded to include nonfluent English speakers and patients with neurodevelopmental disabilities by making translated and accessible versions available.

^a^BCCH: BC Children’s Hospital.

^b^POFU: postoperative follow-up.

## Discussion

### Principal Findings

Our thematic analysis of free-text comments explored parental perspectives on the pediatric surgical journey. Responses to the open-ended prompt captured how they perceived the perioperative and postdischarge experience and their perceptions about the staff and hospital. Over 80% (398/485) of comments were positive or partially positive in sentiment. Key qualitative findings for improvement indicate a need for better organized and uniform communication during the perioperative and postdischarge periods and to set realistic expectations on wakefulness after anesthesia, pain trajectories, and recovery processes. Participant responses also suggested survey amendments. Since over 50% of enrolled participants opted to provide free-text comments, collecting postoperative longitudinal data from caregivers may be a feasible and valuable QI practice for tertiary care facilities.

### Comparison With Previous Work

Our findings confirm and expand on previous work on surgical patient experience conducted at our institution; in an 8-year analysis of narrative data captured as part of the American College of Surgeons National Surgical Quality Improvement Program-Pediatric, Robillard et al [9] reported high rates of satisfaction with the health care experience, but communication and timelines were identified as areas for improvement; they found that parents received adequate information to feel comfortable at the hospital, but not enough information to prepare them for caring for their child at home or what would happen during the healing process. Combined with the present study, these lines of evidence strongly support the value of survey narratives as an effective means of characterizing patient experience.

In follow-up studies using different qualitative methods, such as thematic analyses of audio recordings between parents and nurses at discharge and semistructured parent interviews, the same team uncovered positive experiences with at-home pain management but also highlighted issues of discrepancies in the information provided by different health care providers, and challenges with the timing of discharge information [11,27]. While these similarities support the validity of our findings, the 4-year gap between these 2 studies highlights the challenges in converting these types of data into system-level changes. When multiple stakeholders collaborate to appreciate family perspectives, care providers can create site-specific QI recommendations, as we did following consultation with anesthesiology, nursing, and surgery (Table 3).

Parental feedback has helped inform pediatric surgical guidelines [28,29] and other health care areas [30-33]. Similar to our study, parents indicated they valued the kindness and empathy of health care staff in the neonatal intensive care unit [32]. However, they also desired more effective communication, education, and support [34]. Consistent with our site-specific recommendations (Table 3), improving family-provider communication and integrating purposeful, individualized parental involvement have been strongly recommended to better prepare caregivers for postdischarge care after surgery [35].

### Benefits of Caregiver Perspectives

Each patient’s surgical recovery is unique. In pediatrics, recovery after hospital discharge may be challenging for many reasons; patients may experience pre-existing pain, psychosocial factors of a parent may exacerbate a child’s postoperative pain [13,36], and caregivers with low health literacy may become the primary postsurgical caretakers after hospital discharge [37,38]. The relationship between patients, families, and health care providers is crucial in navigating these complexities. Effective health care involves honoring all perspectives, fostering shared decision-making, and cultivating mutual trust to promote the relationships among patients, families, and providers. Relationship-centered care not only enhances patient satisfaction, adherence to treatment plans, and clinical outcomes but also contributes to provider satisfaction and mitigates burnout [39]. However, implementing individualized care poses challenges, such as time constraints in clinical settings, varying patient and family involvement preferences, and organization-specific barriers.

As we found in this study, gathering family feedback and quantitative data can help uncover areas for QI that may have been underrecognized. Patient stories can also provide a more personal and often more powerful means of reporting positive and negative feedback to perioperative teams. The process of receiving qualitative feedback may remind providers about the importance of communication, foster self-reflection on one’s practices, and further strengthen relationship-centered care.

While implementation of a large-scale hospital- and system-wide change is challenging [40,41], health care facilities should consider implementing routine near real-time initiatives from families (ie, gathering feedback while the patient is in the hospital or shortly thereafter [42]). Electronic technologies have been explored for POFUs in urgent care and emergency departments [43-45] and may be leveraged for QI purposes. As recommended for our institution (Table 3), electronic modalities can streamline communication in both directions—provider to patient and patient to provider. Using e-discharge instructions has successfully improved patient experience outcomes, health care provider workflow [44], and communication between hospitals and primary care providers [46]. Similarly, using e-discharge methods may provide our institution with an opportunity to expand our existing POFU dashboard program [9] to include self-reported free-text feedback. SMS text-messaging or app-based approaches may facilitate patient or family self-reported feedback while improving patient-provider communication [45,47,48] through relationship-centered care.

### Health Care Provider Perspectives

A surgeon is typically the first to discuss postoperative recovery expectations with caregivers and patients. The initial preoperative consultation is essential for establishing a surgeon-family relationship [49], ensuring shared decision-making and the process of informed consent is completed before a surgical procedure is planned. This discussion may occur many months before a procedure takes place (eg, nonurgent elective surgery), maybe in a different care setting (eg, outpatient clinic), may involve a different surgeon (eg, shared waitlists), may need different framing for some recipients (eg, through a translator), and may be received by a different caregiver (eg, with 1 of 2 primary caregivers). These and other factors may impact the information retained and understood, leading to a risk of information conflicting with what other health care providers communicate at later stages of the surgical journey.

In contrast, the surgeon-family relationship differs from the anesthesiologist-family relationship. Patients and caregivers typically meet their anesthesiologist briefly on the day of surgery and may or may not see them again. Consequently, feedback from patients and caregivers about their perioperative experiences is not easily transferred to the anesthesia team. Gathering postdischarge family feedback can support transferring this essential information to healthcare providers and hospital sites (indicating a need for change or confirming the validity of current practices). Our POFU phone calls to families [12, 9], performed by Anesthetic Care Unit nurses, may be enhanced by a perioperative survey scheme similar to the one used in this study. Streamlining communication by advancing our existing POFU program could provide essential quantitative and qualitative feedback for anesthesiologists to improve individual and institutional practice. Natural language processing methods, including large language models, transformers, or latent Dirichlet allocation, or Mamba, may automate content and sentiment analysis to support a continual feedback process [7], reducing the burden on health care staff.

### Limitations

This study is a secondary analysis that addressed a research question that differed from the objectives of the main PPRP study, and thus, our results may be biased. Free-text responses were missed for patients aged 13-18 years during POD 1 to POD 3 due to a study design constraint. The absence of direct patient input represents an overlooked opportunity. Including opportunities for patient and caregiver input at every time point may broaden the family perspective. Furthermore, our participants were recruited from a single institution, potentially limiting the generalizability of our results. Some recommendations we identified will be specific to our institution, but in general, offering families the opportunity to provide free-text comments and using their suggestions to refine perioperative processes should be broadly applicable. In addition, our study attempted to include families with diverse profiles, but non-English speaking families unable to complete the surveys independently and neurodevelopmentally delayed patients were excluded. This representation bias should be addressed for future research endeavors; for example, surveys may use translated versions of validated questionnaires [50], or use REDCap’s multi-language support tool; in addition, the option for verbal survey completion may be incorporated in addition to paper and web-based modalities, which may optimize the collection of responses from families with neurodiverse patients, in particular. Finally, our study process, including in-person recruitment, optional reimbursement incentives for study completion, and manual coding of free-text responses, may not be feasible for large-scale reimplementation of this survey scheme and potentially contributed to a selection bias in our data; nonetheless, the rate of feedback that we received on the optional question suggests that many families may welcome the opportunity to provide free-text feedback.

### Conclusions

The study provides an improved understanding of family perspectives, perceptions, and satisfaction with elements of the pediatric surgical experience. Collecting parental recovery feedback to generate site-specific recommendations in collaboration with health care providers is a feasible and constructive process. Experience data provides useful information to feed into health care providers’ discussion of areas for QI. Tertiary care facilities should consistently and continually engage patients and families, alongside collaboration with other health care providers. Our longitudinal survey instrument may be revised and expanded to support ongoing quality initiatives involving near-real-time patient feedback, thus providing a continual process for improving family-centered care.

## Abbreviations

BCCH: BC Children’s Hospital
CPSP: chronic postsurgical pain
POD: postoperative day
POFU: postoperative follow-up
PPRP: pediatric pain risk prediction
QI: quality improvement
REDCap: Research Electronic Data Capture

## Data Availability

The data sets generated and analyzed during the study are not publicly available due to privacy and ethical requirements, as individual transcripts cannot be shared as per guidance by our research ethics board.
